# Analysis of the status and factors influencing physical activity in patients undergoing ovarian cancer chemotherapy

**DOI:** 10.3389/fonc.2023.1290747

**Published:** 2023-12-13

**Authors:** Shasha Zhang, Fengzhi Zhang, Fangfang Yang, Jimei Yang, Lin Zhang, Junfang Xie

**Affiliations:** ^1^ Department of Gynecology, The Third Affiliated Hospital of Zhengzhou University, Zhengzhou, China; ^2^ Department of Nursing, The Third Affiliated Hospital of Zhengzhou University, Zhengzhou, China; ^3^ Department of Radiotherapy, The First Affiliated Hospital of Zhengzhou University, Zhengzhou, China

**Keywords:** ovarian cancer, physical activity, factors, exercise, fatigue, causality

## Abstract

**Background:**

Ovarian cancer is a common gynecological malignancy, leading to approximately 200,000 deaths globally in 2020. Research has shown that regular physical activity can reduce the toxic side effects of cancer treatment, reduce morbidity and mortality, extend survival time, and improve quality of life. We aimed to evaluate physical activity regimens in patients undergoing chemotherapy for ovarian cancer and analyze the factors influencing physical activity levels.

**Methods:**

To facilitate the selection of patients with ovarian cancer hospitalized for chemotherapy in the Third Affiliated Hospital of Zhengzhou University and the First Affiliated Hospital of Zhengzhou University from August 2022 to February 2023, questionnaire surveys were conducted using the General Information Questionnaire, International Physical Activity Questionnaire, Hospital Anxiety and Depression Scale, and Revised Piper Fatigue Scale.

**Results:**

Data were collected from 167 patients with ovarian cancer. Overall, 96 (57.5%) patients had low physical activity levels, 53 (31.7%) had moderate physical activity levels, and 18 (10.8%) had high physical activity levels. Logistic regression analysis revealed that sleep status, social support, anxiety, depression, and cancer-related fatigue were the main factors influencing physical activity in patients undergoing chemotherapy for ovarian cancer.

**Conclusions:**

Physical activity levels of patients undergoing ovarian cancer chemotherapy were generally low. Therefore, healthcare professionals should pay greater attention to the physical activity in these patients. Targeted and individualized health guidance is recommended, and activity interventions should be implemented according to the influencing factors to promote disease understanding and increase physical activity levels.

## 1 Introduction

Ovarian cancer is a common gynecological malignancy. The latest global cancer statistics for 2020 reported 310,000 new cases of ovarian cancer and 200,000 deaths (1). The incidence and mortality rates of ovarian cancer vary significantly in different regions and countries worldwide but are on a clear upward trajectory in China (2). The disease course of ovarian cancer is insidious and lacks effective screening and early diagnostic measures; as a result, most patients are already in the middle or late stage of the disease at the time of diagnosis. Unfortunately, late-stage ovarian cancer has a poor prognosis, with high recurrence and mortality rates: the 5-year survival rate is less than 20%, and approximately 70% of patients have a recurrence (3, 4). The higher recurrence and mortality rates of ovarian cancer are alarming, and thus, our study was undertaken with the aim of drawing increased attention for this underrepresented patient population. Clinical treatment of ovarian cancer is based on surgery combined with chemotherapy to improve the prognosis. However, treatment side effects significantly impact daily functions, damage physical and mental health, and can even interrupt treatment, endangering the life of the patient (5, 6). As the field of exercise oncology has emerged, many scholars have agreed that exercise has a strong positive effect on the improvement of side effects related to chemotherapy, and that a series of effective behavioral interventions need to be taken to improve exercise adherence. Research has shown that regular physical activity can reduce the toxic side effects of cancer treatment, reduce morbidity and mortality, extend survival time, and improve quality of life (7–9). The American College of Sports Medicine recommends that cancer survivors perform no less than 150 min of moderate-intensity or 75 min of high-intensity physical activity per week (10). Although several international organizations have emphasized that cancer patients can safely exercise during treatment to improve physical functioning and treatment- related complications, this benefit has not been reported in studies of ovarian cancer chemotherapy. Additionally, little is known about the physical activity levels in patients undergoing chemotherapy for ovarian cancer, and it is unclear what factors influence their exercise levels. Therefore, this study aimed to understand the current physical activity status of patients undergoing chemotherapy for ovarian cancer through a cross-sectional survey, analyze the influencing factors to draw the attention of clinical medical personnel to this population’s physical activity patterns, and provide a theoretical basis for the implementation of targeted and individualized interventions.

## 2 Methods

### 2.1 Study participants

Convenience sampling was used to select patients with ovarian cancer who were hospitalized for chemotherapy at the Third Affiliated Hospital of Zhengzhou University and the First Affiliated Hospital of Zhengzhou University between August 2022 and February 2023. Inclusion criteria were as follows: ① Undergoing chemotherapy for a diagnosis of ovarian cancer; ② Age ≥ 18 years; ③ Able to walk independently; ④ No evidence of mental illness or cognitive impairment; ⑤ Willing to voluntarily participate in this study.

The exclusion criteria were: ① Contraindications to activity, such as somatic diseases or severe myelosuppression requiring activity restriction; ② Serious diseases of other systems; ③ Combination of malignant tumors in other parts of the body.

### 2.2 Research tools

#### 2.2.1 General information questionnaire

Based on a review of a large body of literature and in conjunction with our clinical work practices (11–14), a general information questionnaire was designed to collect participant characteristics. The general information questionnaire included age, body mass index, marital status, place of residence, level of education, work status, per capita monthly family income, healthcare payment method, sleep status, complications, tumor stage, number of chemotherapy treatments, and social support.

#### 2.2.2 International physical activity questionnaires

Formulated by the International Physical Activity Measurement Group in 2001 and translated into Chinese by Qu and Li 2004, this questionnaire has a re-test reliability of 0.63-0.89 and validity of 0.60-0.72 (15). The questionnaire assessed the physical activity of the participants in the preceding week, with seven entries involving four levels of physical activity (high-intensity, moderate-intensity, walking, and sitting) assigned a metabolic equivalent of 8.0, 4.0, 3.3, and 1.1, respectively. According to the calculated metabolic equivalent of task values, high physical activity was defined as all kinds of high-intensity physical activity for ≥ 3 days, and overall physical activity level ≥ 1500 metabolic equivalent of task-min/week, or a total of 3 kinds of physical activity for ≥ 7 days, and the overall physical activity level ≥ 3000 metabolic equivalents of task-min/week. Moderate physical activity was defined as all kinds of high-intensity physical activity of at least 20 min per day, for a total of ≥ 3 days, or all kinds of moderate intensity/walking activities of at least 30 min per day, for a total of ≥ 5 days; or physical activity of 3 different types of intensity for a total of ≥ 5 days, and an overall weekly physical activity level ≥ 600 metabolic equivalent of task-min/week. Low physical activity was defined as those who do not meet the requirements for the medium-high subgroup outlined above (16).

#### 2.2.3 Hospital anxiety and depression scale

To assess anxiety and depression in patients with various clinical diseases, this survey is divided into the Self-Assessment Scale for Anxiety (seven items) and the Self-Assessment Scale for Depression (seven items), each of which is scored from 0 to 3 points. A score of 0–7 points represent no symptoms, while 8–10 points represent suspicious symptoms, and 11–21 points represent definite symptoms. The higher the score, the more severe the anxiety and depression. Cronbach’s α coefficients for the total, anxiety, and depression scales were 0.879, 0.806, and 0.806, respectively (17).

#### 2.2.4 Revised piper fatigue scale

The Revised Piper Fatigue Scale was used to measure fatigue symptoms in patients with cancer, including 22 items and three open-ended questions. This method evaluated four dimensions: behavioral fatigue (six items), emotional fatigue (five items), sensory fatigue (five items), and cognitive fatigue (six items). Each entry was scored from 0 to 10 points, with 0 indicating no fatigue and 10 indicating the most severe fatigue. The total score ranged from 0 to 220 points, and the average score was the total score divided by 22. the higher the score, the higher the level of fatigue. Cronbach’s α coefficient for this scale was 0.97 (18).

### 2.3 Data collection

Our study was approved by the Hospital Ethics Committee of the Third Affiliated Hospital of Zhengzhou University (Ethical approval number 2023-097-01). Data collection and analysis were conducted by three postgraduate students with ≥8 years of experience in oncology-related profession and had uniform training. A survey was conducted using a questionnaire during hospitalization. All participants provided informed consent prior to taking the survey. Participants completed the questionnaire independently, and the researchers assisted those with dyslexia or writing difficulties after detailed inquiries using neutral and non-suggestive language.

### 2.4 Statistical analysis

The study included 16 variables and the required sample size was estimated to be 5–10 times the total number of study variables. Considering that 20% of the questionnaires would be invalid, the final required sample size was determined to be 96–192.

Data were analyzed statistically using SPSS 25.0, and quantitative data conforming to normal distribution were expressed as mean ± standard deviation ( $\bar{x}\pm s$ ), and the median and interquartile spacing [M (P25, P75)] were used to express quantitative data with skewed distribution. The qualitative data were expressed as frequency and composition ratio (%). The influential factors were analyzed using the chi-square test, rank-sum test, analysis of variance, and ordered logistic regression analysis. The test level α = 0.05.

## 3 Results

### 3.1 General data of patients undergoing chemotherapy for ovarian cancer

One hundred and seventy-five questionnaires were administered. After exclusion of eight questionnaires with direct data errors and logical errors, 167 valid questionnaires were included in the analysis, with an effective recovery rate of 95.4%. Data from 167 patients undergoing chemotherapy for ovarian cancer with ages ranging from 18 to 78 (51.01 ± 12.26) years were included. Body mass indexes ranged from 16.23 to 30.82 (23.92 ± 2.69) kg/m^2^, the number of chemotherapy treatments ranged from 1 to 12 (3.99 ± 1.94), the anxiety scores were 7.41 ± 3.30, the depression scores were 10.66 ± 3.21, and the fatigue scores were 3.72 ± 1.53. These data and other general information are shown in **Table 1**.

**Table 1:** Comparison of physical activity levels in patients undergoing chemotherapy for ovarian cancer with different characteristics (n=167).

Variant	Number of cases [cases (%)]	Physical activity level			$x^{2}$ */Z*	*p*
		Low (%)	Medium (%)	High (%)		
Age					2.436^b^	0.296
≤ 36 years	25 (15.0)	18 (18.8)	6 (11.3)	1 (5.6)		
36-65 years	132 (79.0)	73 (76.0)	42 (79.3)	17 (94.4)		
> 65 years	10 (6.0)	5 (5.2)	5 (9.4)	0 (0)		
BMI (kg/m^2^)					0.865^b^	0.649
< 18.5	3 (1.8)	1 (1.0)	2 (3.8)	0 (0)		
18.5 to	82 (49.1)	48 (50.0)	27 (50.9)	7 (38.9)		
24.0 to	69 (41.3)	38 (39.6)	20 (37.7)	11 (61.1)		
≥ 28.0	13 (7.8)	9 (9.4)	4 (7.6)	0 (0)		
Place of residence Rural					3.085^a^	0.214
Cities	85 (50.9)	44 (45.8)	29 (54.7)	12 (66.7)		
Towns	82 (49.1)	52 (54.2)	24 (45.3)	6 (33.3)		
Marital status					2.766^a^	0.251
Married	134 (80.2)	73 (76.0)	45 (84.9)	16 (88.9)		
Unmarried, divorced, or widowed	33 (19.8)	23 (24.0)	8 (15.1)	2 (11.1)		
Literacy level					3.962^b^	0.138
Primary and below	63 (37.7)	42 (43.8)	15 (28.3)	6 (33.3)		
Middle school	66 (39.5)	32 (33.3)	22 (41.5)	12 (66.7)		
College and above	38 (22.8)	22 (22.9)	16 (30.2)	0 (0)		
Work status					0.371^a^	0.831
Active	38 (22.8)	22 (22.9)	11 (20.8)	5 (27.8)		
Retired or unemployed	129 (77.2)	74 (77.1)	42 (79.2)	13 (72.2)		
Monthly per capita household income					5.308^b^	0.070
≤ 3000	64 (38.3)	34 (35.4)	24 (45.3)	6 (33.3)		
3000-5000	62 (37.1)	29 (30.2)	22 (41.5)	11 (61.1)		
> 5000	41 (24.6)	33 (34.4)	7 (13.2)	1 (5.6)		
Payment of medical expenses					8.201^a^	0.084
Out-of-pocket expenses	24 (14.4)	10 (10.4)	12 (22.6)	2 (11.1)		
Provincial agricultural cooperative	82 (49.1)	44 (45.8)	26 (49.1)	12 (66.7)		
Municipal/provincial health insurance	61 (36.5)	42 (43.8)	15 (28.3)	4 (22.2)		
Complications					1.401^a^	0.496
None	132 (79.0)	78 (81.3)	39 (73.6)	15 (83.3)		
Yes	35 (21.0)	18 (18.7)	14 (26.4)	3 (16.7)		
Sleep quality					9.638^a^	0.008
Poor	74 (44.3)	52 (54.2)	18 (34.0)	4 (22.2)		
Good	93 (55.7)	44 (45.8)	35 (66.0)	14 (77.8)		
Tumor staging					10.150^b^	0.006
I	26 (15.6)	9 (9.4)	11 (20.8)	6 (33.3)		
II	51 (30.5)	30 (31.2)	13 (24.5)	8 (44.4)		
III	59 (35.3)	40 (41.7)	15 (28.3)	4 (22.3)		
IV	31 (18.6)	17 (17.7)	14 (26.4)	0 (0)		
Number of chemotherapies					2.724^b^	0.256
≤ 3 times	75 (44.9)	40 (41.7)	24 (45.3)	11 (61.1)		
3 to 6 times	78 (46.7)	45 (46.9)	27 (50.9)	6 (33.3)		
> 6 times	14 (8.4)	11 (11.4)	2 (3.8)	1 (5.6)		
Social support					24.814^a^	<0.001
None	106 (63.5)	76 (79.2)	24 (45.3)	6 (33.3)		
Yes	61 (36.5)	20 (20.8)	29 (54.7)	12 (66.7)		

a, $x^{2}$ values; b, Z values; BMI, Body Mass Index.

### 3.2 Physical activity status of patients undergoing ovarian cancer chemotherapy

One hundred sixty-seven patients undergoing ovarian cancer chemotherapy had a daily sedentary time of 212.11 (130, 300) min/day, and a total physical activity metabolic equivalent of 791.55 (330, 1074.45) metabolic equivalent of task-min/week. Among them, 96 (57.5%) had low physical activity levels, 53 (31.7%) had moderate physical activity levels, and 18 (10.8%) had high physical activity levels.

### 3.3 Univariate analysis of physical activity in patients undergoing chemotherapy for ovarian cancer

The univariate analysis showed that the physical activity levels of patients with ovarian cancer for different ages, body mass index, place of residence, marital status, education level, working status, per capita monthly household income, medical expense payment methods, complications, and chemotherapy times were not statistically significant (P > 0.05). In contrast, the physical activity levels of patients with different sleep conditions, tumor stages, and social support were statistically significant (P< 0.05) (**Table 1**).

### 3.4 Correlation analysis of physical activity with sleep quality, tumor staging, social support, anxiety, depression, and fatigue in patients undergoing chemotherapy for ovarian cancer

Physical activity levels were positively associated with sleep quality and social support and negatively correlated with tumor staging, anxiety, depression, and fatigue (**Table 2**). A comparison of the anxiety, depression, and fatigue scores of patients undergoing chemotherapy for ovarian cancer with various levels of physical activity is shown in **Table 3**.

**Table 2:** Correlation of physical activity with sleep quality, tumor staging, social support, anxiety, depression, and fatigue in patients undergoing chemotherapy for ovarian cancer (n=167, r).

Variant	*r*	*P*
Sleep quality	0.240	0.002
Tumor staging	-0.173	0.025
Social support	0.385	<0.001
Anxiety	-0.388	<0.001
Depression	-0.423	<0.001
Fatigue	-0.412	<0.001

**Table 3:** Comparison of anxiety, depression, and fatigue scores in patients undergoing chemotherapy for ovarian cancer with different levels of physical activity (n=167).

Sports event	Physical activity level			*F*	*P*
	Low	Moderate	High		
Anxiety	8.32 ± 3.33	6.58 ± 2.82	5.0 ± 2.72	11.380	<0.001
Depression	11.68 ± 2.30	9.87 ± 2.82	7.56 ± 2.90	17.804	<0.001
Fatigue	4.26 ± 1.47	3.11 ± 1.16	2.62 ± 1.65	17.684	<0.001

### 3.5 Multifactorial analysis of factors influencing physical activity in patients undergoing chemotherapy for ovarian cancer

With physical activity level as the dependent variable, factors with statistically significant differences in univariate analysis (P< 0.05), and anxiety, depression, and fatigue scores were taken as independent variables in the multifactorial logistic regression model for analysis, with the original values of the continuous variables entered and the categorical variables assigned (**Table 4**). This study revealed that sleep quality, social support, anxiety, depression, and fatigue significantly influenced the physical activity levels of patients undergoing ovarian cancer chemotherapy (P< 0.05) (**Table 5**).

**Table 4:** Assignment of variables.

Variant	Variable assignment
Physical activity level	Low = 1; Moderate = 2; High = 3
Sleep quality	Better = 1; Worse = 2
Tumor staging	I=1; II=2; III=3; IV=4
Social support	Yes = 1; No = 2
Anxiety	Continuous variables
Depression	Continuous variables
Fatigue	Continuous variables

**Table 5:** Ordered logistic regression analysis of factors influencing physical activity in patients undergoing chemotherapy for ovarian cancer (n=167).

Independent variable	*β*	*SE*	Wald $x^{2}$	*P*	*OR*	*95% CI*
Sleep quality	1.082	0.396	7.455	0.006	2.950	1.357-6.412
Social support	1.501	0.388	14.948	<0.001	4.486	2.096-9.600
Anxiety	-0.154	0.072	4.502	0.034	0.858	0.744-0.988
Depression	-0.250	0.075	11.020	0.001	0.779	0.672-0.903
Fatigue	-0.451	0.138	10.733	0.001	0.637	0.486-0.834

SE, standard error; OR, odds ratio; CI, confidence interval.

## 4 Discussion

Physical activity levels urgently need to be increased in patients with ovarian cancer undergoing chemotherapy. In this study, over half of the patients had a low physical activity level, which is consistent with previous studies (19). Only 25.1% of the patients in this study achieved the guideline-recommended amount of exercise, which is slightly higher than that in the study by Mizrahi et al. (12) this is related to the regional environment and the exercise benefits of recent years, but the overall physical activity levels remain low. Jones et al. (14) noted in a systematic review of physical activity in survivors of ovarian cancer that most patients had reduced physical activity after a clear diagnosis. This suggests that the physical activity level of patients undergoing chemotherapy for ovarian cancer both at home and abroad is insufficient and needs to be improved. The underlying reasons for reduced physical activity may be as follows: first, the side effects of nausea, vomiting, and fatigue during chemotherapy consume most of the patient’s physical strength, which affects the orderly conduct of activities; second, postoperative recovery time in some patients is relatively short, and their optimal state of fitness was not reached, thus decreasing the number of activities; third, during the hospitalization period, the lack of space and variety for fitness and sports facilities due to the limited environment affects the activities to a certain extent. To address this situation, medical personnel should attach great importance to physical activity in these patients. On the one hand, they should efficiently manage the side effects of treatment, strengthen disease education, and improve the traditional misperception of sedation. On the other hand, based on the wide range of benefits of exercise for patients with cancer, medical personnel may consider incorporating exercise into clinical practice as a part of clinical treatment programs. Moderate-intensity physical activity regulates cardiovascular function and can be performed daily (20). However, high-intensity physical activity is also a safe, effective, and feasible clinical intervention for patients with cancer. It can effectively inhibit tumor growth, improve cardiorespiratory function and health, and reduce detrimental effects such as fatigue (21, 22). Therefore, in clinical practice, medical personnel should provide patients with targeted and individualized guidance on high-intensity physical activity within the patient’s tolerability. Finally, medical institutions at all levels should establish and improve relevant exercise facilities and venues to provide patients with an exercise-positive environment.

Several factors influenced the physical activity levels in our study:

(1) Sleep quality.

The regression analysis unveiled a notable statistical association between sleep quality and physical activity levels (OR=2.950, 95% CI 1.357-6.412, P=0.006). Specifically, compared to patients with poorer sleep quality, patients with better sleep quality were2.950 times more likely to increase their physical activity level by one grade. The physical activity level of patients with better sleep quality was relatively high, which is consistent with previous studies (23, 24). Sleep is an essential process in physical and psychological recovery, and adequate sleep is conducive to maintaining body energy and desire to perform physical activity. Moreover, appropriate moderate-to-high-intensity physical activity also helps improve sleep quality (25). This suggests that healthcare professionals in clinical practice, in addition to creating a quiet, comfortable sleep environment, should encourage patients to exercise during the day to improve sleep quality by ensuring adequate sleep at night.

(2) Social support.

The regression analysis showed a statistically significant relationship between social support and physical activity level (OR=4.486,95% CI 2.096-9.600, P<0.001). Specifically, compared to patients without social support, patients with social support were 4.486 times more likely to increase their physical activity level by one grade. Physical activity level was better in patients with social support, which is in line with the results of Huang et al. (26). Additionally, Smith et al. (27) found that support from family members led to more leisure time and physical activity. This may be because encouragement from family and friends and guidance from healthcare professionals can alleviate psychological burden and stimulate their motivation to exercise, improving exercise compliance. This suggests that healthcare professionals should strengthen caregivers’ exercise rehabilitation education while encouraging patients to actively exercise and work together to formulate personal exercise programs suitable for patients of all skill levels.

(3) Anxiety and depression.

Anxiety and depression are risk factors for reduced physical activity levels in patients undergoing chemotherapy for ovarian cancer. For each point increase in the anxiety or depression score, the probability of patients increasing physical activity level by one grade was 0.858 and 0.779 times higher than before, respectively. The more severe the anxiety and depression, the lower the level of physical activity. Teychenne et al. (28) concluded that a depressed mood increases the impairment in physical activity. Helgadóttir et al. (29) found that patients with anxiety and depression were more inclined to engage in sedentary behavior. This may be due to the patient’s unwillingness to exercise or fear of activity due to the loss of ovarian function after surgical removal of the ovaries, decreased secretion of sex hormones, or depressed mood resulting from endocrine dysfunction. A previous study showed that the more fearful patients were of activity, the more likely they were to develop sedentary behavior (30). In general, female patients with cancer tend to be more emotionally sensitive and vulnerable, have weak psychological resilience, poor self-regulation, and exhibit higher levels of anxiety and depression (31). Compared with other cancer populations, women treated for ovarian cancer are at a higher risk of anxiety and depression (32), with frequent negative emotions, and lack of motivation to exercise. This suggests that healthcare professionals should assess patients’ psychological conditions in real-time, establish effective communication, divert patients’ attention, provide psychological support, allow patients to establish confidence in overcoming the disease, and alleviate negative emotions to stimulate motivation to exercise.

(4) Cancer-related fatigue.

The regression analysis showed that for each point increase in the fatigue score, the probability of patients increasing their physical activity level by one grade was 0.637 times higher than before. Regression analysis revealed a negative correlation between fatigue and physical activity level, which is consistent with the results of previous studies (12). A prospective study by Eyl et al. (33) found that physical activity in patients with cancer was closely related to decreased fatigue. Fatigue is one of the most common and unmanageable symptoms in patients with cancer, with an incidence rate of 30%–99% (34, 35). Patients with fatigue have a self-perceived depletion of energy and stamina, and decreased interest in exercise leads to reduced physical activity. Exercise is the most effective non-pharmacological intervention for reducing fatigue, and several international organizations have emphasized the safety and importance of exercise in treating patients with cancer (10, 36–38). Therefore, healthcare professionals should understand why patients do not exercise, explain the importance of exercise and the mechanisms by which exercise changes mood and energy, and adopt practical exercise strategies to encourage patients to actively participate in exercise to alleviate the side effects of treatment.

## 5 Conclusion

The physical activity levels of patients receiving chemotherapy for ovarian cancer are low and need to be improved. Poor sleep, lack of social support, anxiety, depression, and cancer-related fatigue were influencing factors. Healthcare professionals should promptly assess the health of patients with these risk factors and provide targeted health guidance based on the specific conditions of the patients. Practical and effective interventions should be used to encourage these individuals to actively engage in various types of physical activities. This will not only improve their activity levels but also promote overall health improvement.

## 6 Limitations of this study

Firstly, we specifically examined patients with ovarian cancer undergoing chemotherapy in two hospitals, which could affect the generalizability of our findings due to the restricted sample size and representativeness. Moreover, the influence of other factors possibly related to physical activity, such as nutrition, pain, and wound healing, may have been overlooked. A multicenter and large-sample study may be carried out in the future that incorporates other important factors affecting the physical activity of patients with ovarian cancer undergoing chemotherapy. Additionally, the use of self-reported physical activity questionnaires may have led to bias, and the level of physical activity may not have been accurately quantified. To further validate our findings, future investigations could integrate objective tools and assessments for data collection and consider conducting longitudinal studies. Finally, the results of this study focused on the isolated impact of individual variables on the level of physical activity. The interactions between variables may have an impact on the level of physical activity, which warrants further exploration in future studies.

## 7 Implications for clinical medical personnel

Our study has strong practical implications for clinical work and offers important guidance recommendations, emphasizing the need for future interventions in physical activity in patients undergoing chemotherapy for ovarian cancer to improve physical activity levels and thus treatment-related complications. In addition, medical workers should receive relevant professional training to fully assess the factors affecting the physical activity level of patients undergoing chemotherapy for ovarian cancer and adopt effective strategies to improve the physical activity level of patients. One final recommendation is that clinical workers create a positive and supportive exercise environment for patients. They should design targeted and individualized exercise programs based on the full evaluation of the patients’ physical and mental conditions, with the view to improve the adherence to physical activity and ultimately the quality of life of patients.

## Data availability statement

The raw data supporting the conclusions of this article will be made available by the authors, without undue reservation.

## Ethics statement

The studies involving humans were approved by the Ethics Committee of the Third Affiliated Hospital of Zhengzhou University. The studies were conducted in accordance with the local legislation and institutional requirements. The participants provided their written informed consent to participate in this study.

## Author contributions

SZ: Conceptualization, Data curation, Formal Analysis, Investigation, Software, Writing – original draft. FZ: Funding acquisition, Project administration, Resources, Supervision, Writing – review & editing, Data curation. FY: Conceptualization, Formal Analysis, Investigation, Software, Writing – review & editing. JY: Investigation, Resources, Writing – review & editing. LZ: Investigation, Writing – original draft, Data curation, Formal Analysis. JX: Supervision, Writing – review & editing.
